# Thymic Alterations in GM2 Gangliosidoses Model Mice

**DOI:** 10.1371/journal.pone.0012105

**Published:** 2010-08-10

**Authors:** Seiichi Kanzaki, Akira Yamaguchi, Kayoko Yamaguchi, Yoshitsugu Kojima, Kyoko Suzuki, Noriko Koumitsu, Yoji Nagashima, Kiyotaka Nagahama, Michiko Ehara, Yoshio Hirayasu, Akihide Ryo, Ichiro Aoki, Shoji Yamanaka

**Affiliations:** 1 Department of Pathology, Yokohama City University School of Medicine, Yokohama, Japan; 2 Department of Microbiology, Yokohama City University School of Medicine, Yokohama, Japan; 3 Department of Psychiatry, Yokohama City University School of Medicine, Yokohama, Japan; Massachusetts General Hospital and Harvard Medical School, United States of America

## Abstract

**Background:**

Sandhoff disease is a lysosomal storage disorder characterized by the absence of β-hexosaminidase and storage of GM2 ganglioside and related glycolipids. We have previously found that the progressive neurologic disease induced in *Hexb* ^−/−^ mice, an animal model for Sandhoff disease, is associated with the production of pathogenic anti-glycolipid autoantibodies.

**Methodology/Principal Findings:**

In our current study, we report on the alterations in the thymus during the development of mild to severe progressive neurologic disease. The thymus from *Hexb* ^−/−^ mice of greater than 15 weeks of age showed a marked decrease in the percentage of immature CD4^+^/CD8^+^ T cells and a significantly increased number of CD4^+^/CD8^−^ T cells. During involution, the levels of both apoptotic thymic cells and IgG deposits to T cells were found to have increased, whilst swollen macrophages were prominently observed, particularly in the cortex. We employed cDNA microarray analysis to monitor gene expression during the involution process and found that genes associated with the immune responses were upregulated, particularly those expressed in macrophages. CXCL13 was one of these upregulated genes and is expressed specifically in the thymus. B1 cells were also found to have increased in the thy mus. It is significant that these alterations in the thymus were reduced in *FcRγ* additionally disrupted *Hexb* ^−/−^ mice.

**Conclusions/Significance:**

These results suggest that the FcRγ chain may render the usually poorly immunogenic thymus into an organ prone to autoimmune responses, including the chemotaxis of B1 cells toward CXCL13.

## Introduction

Lysosomal storage disorders (LSDs) arise from functional defects in one or more of the proteins essential to normal lysosome function. This typically involves the enzymes that play a critical role in the intracellular digestion of glycoproteins, glycolipids, glycosaminoglycans, or other macromolecules [1]. GM2 gangliosidoses, one of the major LSDs, are caused by an abnormality in the β-hexosaminidases [1], [2]. β-Hexosaminidase A consists of a heterodimer of an α-subunit (*HEXA* gene product) and a β-subunit (*HEXB* gene product) whereas β-Hexosaminidase B is a homodimer of β-subunits. Mutations in the *HEXA* gene cause Tay-Sachs disease, whereas mutations in the *HEXB* gene cause Sandhoff disease (SD) [1].

Previous studies have shown that the *Hexb*-deficient (*Hexb* ^−/−^) mouse develops an SD-like illness and therefore provides a useful animal model for investigating the pathophysiology of SD [3]–[5]. As with many of the other LSDs, neurodegeneration is a prominent feature of SD and since the neurons accumulate a large amount of GM2 ganglioside and GA2 relative to other tissues in this disease, it is generally thought that the nervous system is its main pathological target. The accumulation of GM2 ganglioside or its derivatives in the nervous system is implicated in unscheduled neuronal cell death [6]. However, recent studies have provided good evidence that GM2 ganglioside and GA2 accumulation can not account for all of the nervous system damage in *Hexb* ^−/−^ mice.

For example, bone marrow transplantation from normal (*Hexb^+/+^*)to *Hexb* ^−/−^ mice suppresses neuronal death and improves survival ratios despite having no effect on either β-hexosaminidases activities or ganglioside accumulation in the brain [7]–[9].

We have reported in a previous study that an autoimmune response occurs in *Hexb* ^−/−^ mice with accompanying pathophysiological phenotypes [10]. To determine the role of anti-ganglioside autoantibodies in this earlier study, we additionally disrupted the Fc receptor gamma (*FcRγ*) gene in the *Hexb^−/−^* mouse model, as it plays a key role in immune complex-mediated autoimmune diseases. Clinical symptoms were improved and life spans were extended in the resulting double-null (*Hexb* ^−/−^ *FcRγ* ^−/−^)mice, suggesting that autoantibodies play an important role in the central nervous system (CNS) pathophysiology in SD. Moreover, we found that age-matched *Hexb^−/−^FcRγ^−/−^* mice had a reduced serum titer of anti-GA2 autoantibody when compared with *Hexb* ^−/−^ *FcRγ^+/+^* mice, suggesting that the FcRγ dependent pathway(s) also contribute to the production of autoantibodies [10]. Recently also, autoantibodies have been implicated in several LSDs and their respective mouse models such as MPSIIIB [11], Batten disease [12]–[15], and Gaucher disease [16], [17]. This suggests that the production of autoantibodies is mediated by common pathway(s) among these diseases although the underlying mechanism of production remains unknown.

The thymus is a central or primary lymphoid organ responsible for the production, differentiation and direction of a population of small lymphocytes that are involved primarily with cell-mediated immunity. Such thymus-dependent lymphocytes are known as T-lymphocytes in contrast to the bone marrow dependent B-lymphocytes with which they intimately co-operate. Recently, alterations in the thymus were observed in several LSD model animals such as feline GM1 gangliosidosis [18]–[20], and twitcher mice [21]. In *Hexb^−/−^* mice, GM2 and GA2 were found to have accumulated in the thymus [22]. Impaired selection of invariant natural killer T cells was also observed in this same study [22]. However, whether such alterations in the thymus contribute to the LSD pathophysiology remains unknown.

Over the last decade, a family of chemotactic cytokines known as the chemokines has been found to be involved in the trafficking of leukocytes in both normal and pathological states. Several autoimmune diseases, such as rheumatoid arthritis, systemic lupus erythematosus, multiple sclerosis, and myasthenia gravis, are associated with inappropriate activation of the chemokine network [23]. For example, aberrant high expression of Chemokine (C-X-C motif) ligand 13 (CXCL13) in the thymus in aged (NZB × NZW) F1 (BWF1) mice may play a pivotal role in breaking immune tolerance in the thymus and in recruiting autoantibody-producing B1 cells and CD4^+^T cells during the development of murine lupus [24]–[26]. B1 cells of different origin and function than conventional B cells (B2 cells) have long been considered to be involved in autoantibody production in autoimmune diseases [27]–[29].

In *Hexb* ^−/−^ mice, cDNA microarray analysis of the CNS has previously revealed an upregulation of inflammatory cytokines/chemokines including CXCL13, dominated by activated microglia/macrophages [8]. The expected mechanism underlying this microglia/macrophage activation was the storage of glycolipids from engulfed apoptotic neurons. Normally, most T cells are eliminated within the cortex or at the cortico-medullary junction via programmed cell death. These T cells are usually rapidly engulfed by macrophages without accompanying immune activation. It is thus possible that if the macrophages cannot degrade apoptotic T cell-derived glycolipids, they are also activated.

We hypothesize that the thymus plays an important role in the production of autoantibodies in SD. We have therefore examined the thymic alterations of *Hexb* ^−/−^ mice during the development of mild to severe progressive neurologic disease in our current study and explored the relationship between these thymic abnormalities and autoimmunity.

## Results

### *Hexb* ^−/−^ *FcRγ* ^+/+^ mice develop progressive and age-related thymus atrophy

**Figure 1:**
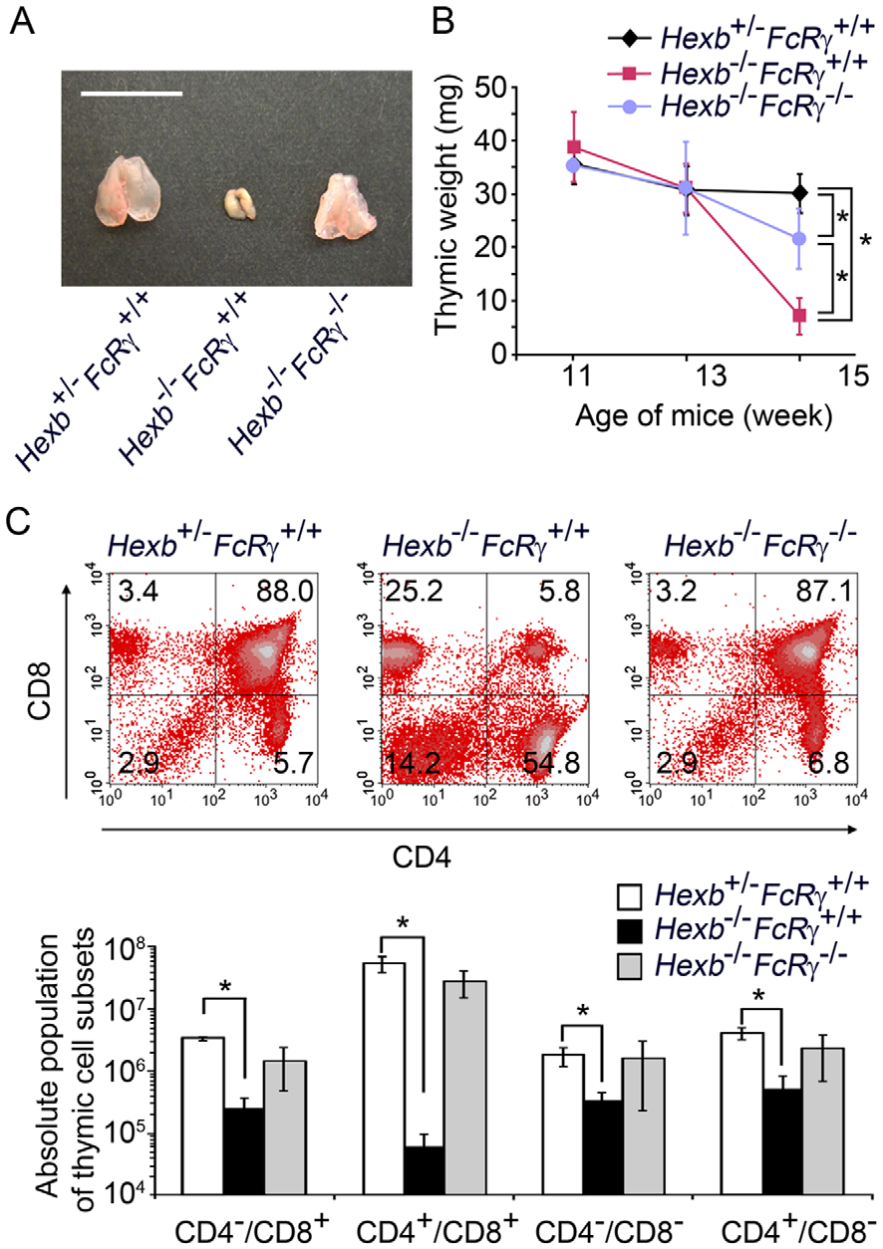
*Hexb* ^−/−^ *FcRγ* ^+/+^ mice show progressive, age-related thymus atrophy. (A) The thymus was removed from 15 week old *Hexb* ^+/−^ *FcRγ^+/+^*, *Hexb* ^−/−^ *FcRγ^+/+^* and *Hexb* ^−/−^ *FcRγ* ^−/−^ mice. Scale bar, 1 cm. (B) Thymic tissues from *Hexb* ^+/−^ *FcRγ^+/+^*, *Hexb* ^−/−^ *FcR^+/+^* and *Hexb* ^−/−^ *FcR* ^−/−^ mice at 11, 13 and 15 weeks of age were removed and weighed. The data shown are expressed as the mean (S.D. of 3–14 mice for each group per time point. *P<0.01. (C) Thymic cells from *Hexb* ^+/−^ *FcR* ^+/+^, *Hexb* ^−/−^ *FcR* ^+/+^ and *Hexb* ^−/−^ *FcR* ^−/−^ mice were stained with CD4 and CD8 antibodies. The percentages of T cell subsets were then determined by flow cytometry. The profiles for each genotype are indicated in the top panels. The absolute cell numbers for each subpopulation of the thymus were calculated from the total number of thymic cells (mean (S.D.; *n* = 3–4). *P<0.05.

Our initial analysis indicated that *Hexb* ^−/−^ *FcRγ^+/+^* mice with clinically severe neurologic signs had a severely reduced thymic mass (Fig. 1A). In comparison, age-matched *Hexb^−/−^FcRγ^−/−^* mice had only a slight reduction in their thymic mass. To evaluate whether these thymic alterations were due to hypoplasia or premature involution, thymic tissues from younger mice were also examined quantitatively. A comparison of *Hexb* ^+/−^ *FcRγ^+/+^*, *Hexb* ^−/−^ *FcRγ^+/+^* and *Hexb^−/−^FcRγ^−/−^* thymic weights is shown in Figure 1B. The 15 week old *Hexb* ^−/−^ *FcRγ^+/+^* mice showed significant (75%) loss of thymic weight when compared with age-matched *Hexb* ^+/−^ *FcRγ^+/+^* mice. In contrast, there were no significant differences between the younger mice of both genotypes. *Hexb^−/−^FcRγ^−/−^* mice had a slight, but statistically significant, reduction in thymic weight when compared with *Hexb* ^+/−^ *FcRγ^+/+^* mice. Flow cytometric analysis further revealed that CD4^+^/CD8^+^ immature T cell ratio was dramatically decreased in the 15 week old *Hexb* ^−/−^ *FcRγ^+/+^* thymus compared with that of the *Hexb^+/−^FcRγ^+/+^* mice (5.8% vs. 88.0%), whereas the CD4^+^/CD8^−^ and CD4^−^/CD8^+^ T cell ratios were markedly increased (Fig. 1C top panel). Moreover, the CD4^−^/CD8^−^ non T cell population ratio was also increased in *Hexb* ^−/−^ *FcRγ^+/+^* mice. In *Hexb^−/−^FcRγ^−/−^* mice, the percentages of CD4^+^/CD8^+^, CD4^+^/CD8^−^, CD4^−^/CD8^+^, and CD4^−^/CD8^−^ cells were 87.1%, 6.8%, 3.2%, and 2.9%, respectively. The absolute number of CD4^+^/CD8^+^ T cells per thymus was severely reduced in *Hexb* ^−/−^ *FcRγ^+/+^* mice (Fig. 1C bottom panel). In addition, the absolute number of CD4^+^/CD8^−^, CD4^−^/CD8^+^, and CD4^−^/CD8^−^ T cells per thymus was also reduced in *Hexb* ^−/−^ *FcRγ^+/+^* mice.

### Histological examination of the thymus from *Hexb* ^+/−^ *FcRγ* ^+/+^, *Hexb* ^−/−^ *FcRγ* ^+/+^ and *Hexb* ^−/−^ *FcRγ* ^−/−^ mice

**Figure 2:**
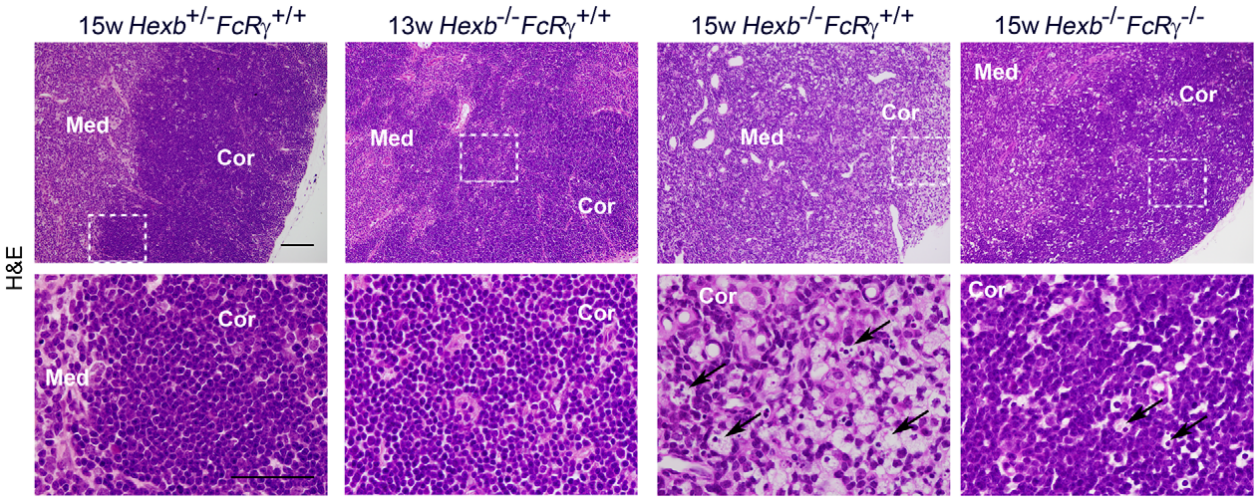
Histological examination of the thymus from *Hexb* ^+/−^ *FcRγ* ^+/+^, *Hexb* ^−/−^ *FcRγ* ^+/+^ and *Hexb* ^−/−^ *FcRγ* ^−/−^ mice. H&E staining of paraffin-embedded thymic sections from 13 week old *Hexb* ^+/−^ *FcRγ^+/+^*, 15 week old *Hexb* ^+/−^ *FcRγ^+/+^*, *Hexb* ^−/−^ *FcRγ^+/+^* and *Hexb* ^−/−^ *FcRγ* ^−/−^ mice. The bottom panels show a higher magnification image of the framed area in the top panels. Cor, cortex; Med, medulla. Arrows indicate vacuolated cells. Scale bars, top panel, 100 µm; bottom panel, 50 µm.

We next undertook histological analysis of the thymic tissues from *Hexb* ^+/−^ *FcRγ^+/+^*, *Hexb* ^−/−^ *FcRγ^+/+^* and *Hexb^−/−^FcRγ^−/−^* mice. When examined by light microscopy, the histological findings from younger *Hexb* ^−/−^ *FcRγ^+/+^* mice were found not to significantly differ from *Hexb* ^+/−^ *FcRγ^+/+^* mice. In the 15 week old *Hexb* ^−/−^ *FcRγ^+/+^* mice, the cellular density did not appear to be altered in the medulla of the thymus but the cortices or cortico-medullary junctions were frequently indistinct (Fig. 2). Moreover, many foamy macrophages could be observed not only in the medullary area, but also the cortex area in these mice. Some macrophages were also found to contain hematoxylin-positive nuclear particles in their cytoplasm. Cortices or cortico-medullary junctions were clearly detectable in the thymus of *Hexb^−/−^FcRγ^−/−^* mice and foamy macrophages were also present in *Hexb^−/−^FcRγ^−/−^*mice. We also examined the immunofluorescent analysis of GA2 and thymocyte, for each thymus derived from *Hexb* ^+/−^ *FcRγ^+/+^*, *Hexb* ^−/−^ *FcRγ^+/+^* and *Hexb* ^−/−^ *FcRγ^−/−^* mice. In the 15 week old *Hexb* ^+/−^ *FcRγ^+/+^* mice, GA2 was stained on the cell membrane of CD4 and/or CD8 positive T cells (Fig. S1). Age matched *Hexb* ^−/−^ *FcRγ^+/+^* mice showed the marked decrease of the T cells in the cortex area. In contrast, GA2 was stained in those areas, and it seems to be accumulating in the cytosol of the foamy cells. The decrease of the T cell and the accumulation of GA2 were not more clearly observed in the presymptomatic stage of *Hexb* ^−/−^ *FcRγ^+/+^*, and *Hexb* ^−/−^ *FcRγ^−/−^* mice. These results suggest the possibility that the localization of GA2 was moved from T cells to foamy macrophages during the involution.

### Histological examination of a thymus from a human SD patient

**Figure 3:**
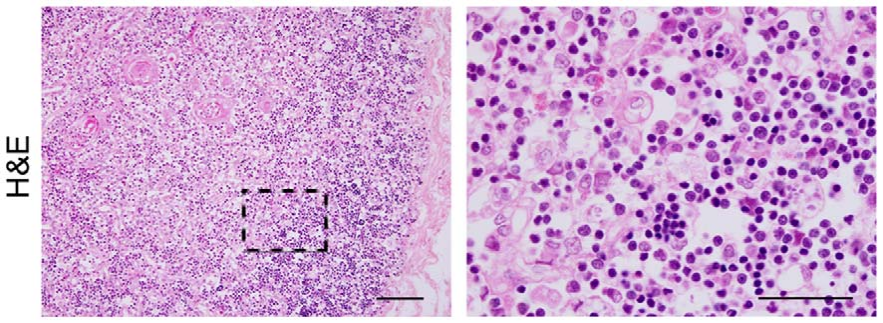
Histological examination of a thymus from an SD patient. H&E staining of a paraffin-embedded thymic section from an 11 month-old SD patient. The right panel shows a higher magnification of the framed area in the left panel. Scale bars, left panel, 100 µm; right panel, 50 µm.

To determine whether thymic alterations can also be detected in a human case of SD, we obtained a H&E stained tissue sample from an SD patient for examination. The histology of this thymic sample revealed that the cortical cells were severely reduced and that the cortico-medullary junctions could not be clearly observed (Fig. 3). Furthermore, most of the cortical and medullary cells harbored a vacuolated cytoplasm.

### Increased cell death in the thymus of 15 week old *Hexb* ^−/−^ *FcRγ* ^+/+^ mice

**Figure 4:**
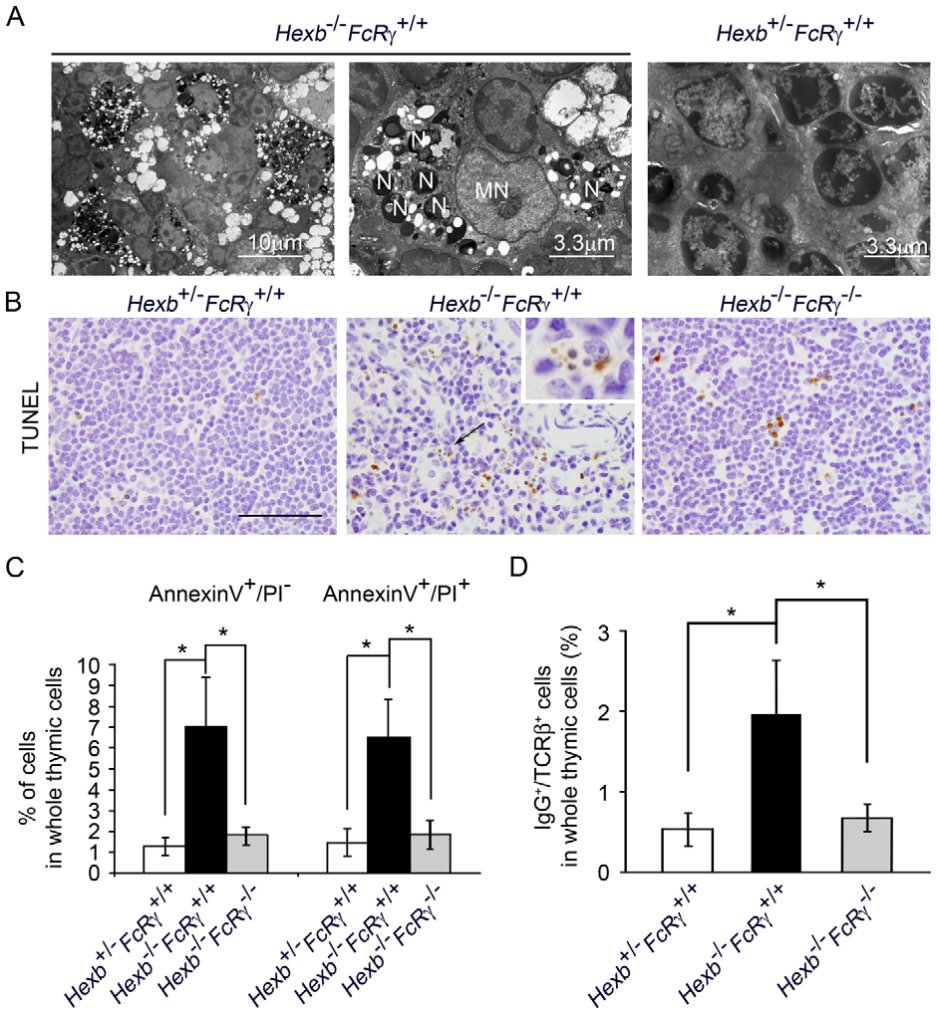
Increased cell death in the thymus of the 15 week old *Hexb* ^−/−^ *FcRγ* ^+/+^ mouse. (A) Thymic sections from 15 week old *Hexb* ^−/−^ *FcRγ^+/+^* and *Hexb* ^+/−^ *FcRγ^+/+^* mice were analyzed by TEM. Left panel, at lower magnification many electron dense materials and vacuoles are evident. Middle panel, nucleus-like electron dense particles and vacuoles are visible in the cytoplasm of the macrophages at higher magnification. Right panel, nuclear particles were not confirmed in the thymus of the *Hexb* ^+/−^ *FcRγ^+/+^* mouse.N, nucleus particle-like structure; MN, macrophage nucleus. (B) Paraffin-embedded thymic sections from *Hexb* ^+/−^ *FcRγ^+/+^*, *Hexb* ^−/−^ *FcRγ^+/+^* and *Hexb* ^−/−^ *FcRγ* ^−/−^ mice were stained using the TUNEL method. Scale bar, 50((m. A higher magnification image of the arrow position in the middle panel is shown in the right shoulder frame. (C) Thymic cells from *Hexb* ^+/−^ *FcR^+/+^*, *Hexb* ^−/−^ *FcR^+/+^* and *Hexb* ^−/−^ *FcR* ^−/−^ mice were stained with FITC conjugated anti-Annexin V antibody and PI, and the percentages of dying cells were determined by flow cytometry (*n* = 4–8). *P<0.01. (D) IgG deposition in TCR(-positive T cells analyzed by flow cytometry (*n* = 3–5). *P≤0.01.

The thymic tissues from 15 week old *Hexb* ^+/−^ *FcRγ^+/+^* and *Hexb* ^−/−^ *FcRγ^+/+^* mice were further analyzed by transmission electron microscopy (TEM). In the *Hexb* ^−/−^ *FcRγ^+/+^* mice, the macrophages contained numerous abnormal nuclear-like electron dense particles, which were not evident in the *Hexb* ^+/−^ *FcRγ^+/+^* thymus (Fig. 4A). These macrophages were also found to contain many vacuoles with characteristics of lysosomal storage vessels. To better understand the progressive reduction observed in the number of thymic cells, we analyzed thymic cell death using a TUNEL assay. TUNEL-positive cells were more frequently found in the *Hexb* ^−/−^ *FcRγ^+/+^* cortex and medulla than in the corresponding regions of the *Hexb* ^+/−^ *FcRγ^+/+^* thymus (Fig. 4B). TUNEL-positive particles could be seen inside the macrophages, and were thought to correspond to the nuclear-like particles observed by TEM. We next quantified the number of apoptotic and necrotic cells by flow cytometry. The results showed that the numbers of AnnexinV^+^/PI^−^ apoptotic cells in *Hexb* ^+/−^ *FcRγ^+/+^*, *Hexb* ^−/−^ *FcR^+/+^* and *Hexb* ^−/−^ *FcR* ^−/−^ mice were 1.28±0.44, 7.06±2.32, and 1.79±0.45 respectively. Moreover, AnnexinV+/PI+ late apoptotic/necrotic cells were found to have increased in the *Hexb* ^−/−^ *FcR^+/+^* thymus (1.47±0.68, 6.45±1.87, and 1.85±0.69, respectively; Fig. 4C). Since thymic cell death in the *Hexb^−/−^FcR* ^−/−^ mice is decreased, we hypothesized that the immune-complex mediated response is responsible for accelerating thymic cell death in *Hexb* ^−/−^ *FcR^+/+^* mice. Indeed, we found a marked increase in the percentage of IgG/TCR-(double positive T cells (0.53±0.20% vs. 1.96±0.67%), suggesting that IgG is bound to the T cell membrane, in the 15 week old *Hexb* ^−/−^ *FcR^+/+^* mice as compared with age-matched *Hexb* ^+/−^ *FcR^+/+^* mice. The 15 week old *Hexb^−/−^FcR^−/−^* mice also showed a reduction in this population of cells (Fig. 4D).

### Macrophage activation and expansion in thymus

**Figure 5:**
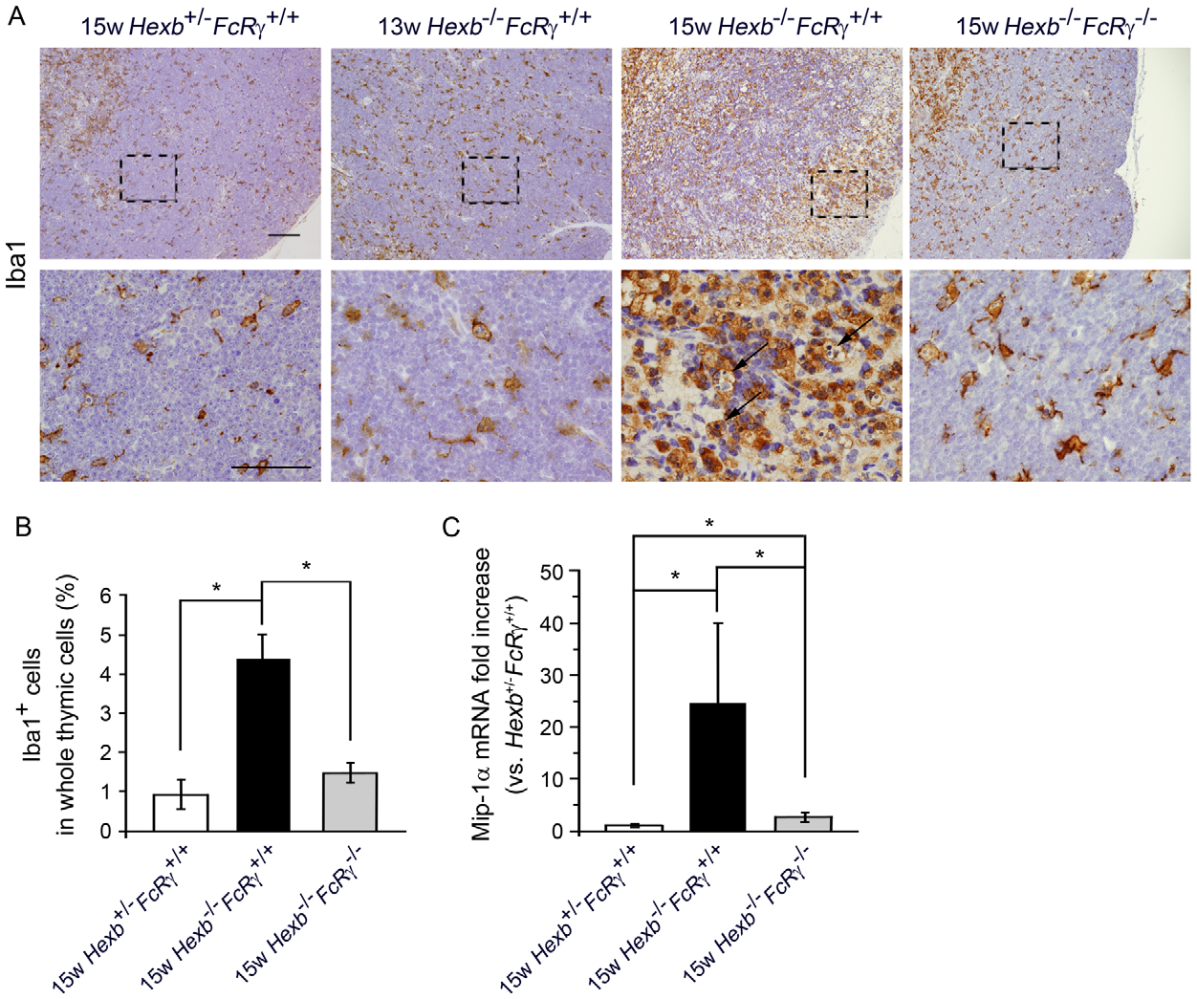
Macrophage activation and expansion. (A) Thymic tissues from 13 week old *Hexb* ^+/−^ *FcR^+/+^*, 15 week old *Hexb* ^+/−^ *FcR^+/+^*, *Hexb* ^−/−^ *FcR^+/+^* and *Hexb* ^+/−^ *FcR^+/+^* mice were stained with antibodies against the macrophage marker Iba1 and processed using the avidin-biotin-peroxidase complex (ABC) method. The bottom panels show higher magnifications of the framed areas in the top panels. Scale bars, top panel, 100(µm; bottom panel, 50 µm. (B) Thymic cells from15 week old *Hexb* ^+/−^ *FcRγ^+/+^*, *Hexb* ^−/−^ *FcRγ^+/+^* and *Hexb* ^−/−^ *FcRγ* ^−/−^ mice were stained with anti-Iba1 followed by Alexa Fluor 488® conjugated anti-rabbit IgG (H+L), and the percentages of macrophages were determined by flow cytometry (*n* = 4). *p<0.01. (C) Mip-1α mRNA levels in the thymus from 15week old *Hexb* ^−/−^ *FcRγ^+/+^* and *Hexb* ^−/−^ *FcRγ* ^−/−^ mice were assayed by Real-time RT-PCR and these values were compared with those of the *Hexb* ^+/−^ *FcRγ^+/+^* mouse (mean ± S.D.; *n* = 5–9). *P<0.05.

The swelling of macrophages is a prominent feature of many LSDs. To confirm whether the swollen cells we observed by H&E staining of our thymic sections were macrophages, we stained these samples with Iba-1 and found that positive cells were rarely observed in the cortices of the 15 week old *Hexb* ^+/−^ *FcR^+/+^* mice. 13 week old *Hexb* ^−/−^ *FcR^+/+^* mice were found not to significantly differ from *Hexb* ^+/−^ *FcRγ^+/+^* mice. On the other hand, numerous Iba-1 positive swelling cells were detected in the 15 week old *Hexb* ^−/−^ *FcR^+/+^* thymus, particularly in the cortex. In *Hexb^−/−^FcR^−/−^* mice, at 15 weeks of age, the numbers of both foamy cells and amoeboid, Iba-1-positive thymic macrophages were substantially reduced compared with the *Hexb^−/−^FcR^+/+^* mice (Fig. 5A). To determine whether the percentage of macrophages among the total thymic cell population was increased, we performed flow cytometric analysis. The percentage of thymic macrophages from 15 week old *Hexb* ^+/−^ *FcR^+/+^*, *Hexb* ^−/−^ *FcR^+/+^* and *Hexb* ^−/−^ *FcR* ^−/−^ mice was 0.93±0.38, 4.36±0.66 and 1.48±0.25% respectively (Fig. 5B). Recent studies have reported that the up-regulation of the Mip-1α correlates with monocyte infiltration and the pathogenesis of SD [30]. To quantify the increase in Mip-1α mRNA in the thymus of 15 week old *Hexb* ^−/−^ *FcRγ^+/+^* mice, the gene expression ratios of Mip-1α relative to ribosomal protein s18 were measured by real-time RT-PCR. A 24.6-fold increase in Mip-1α expression was detected in the thymus of *Hexb* ^−/−^ *FcRγ^+/+^* mice in comparison with *Hexb* ^+/−^ *FcRγ^+/+^* mice. In addition, *Hexb^−/−^FcRγ^−/−^* mice showed a 2.69-fold higher expression of Mip-1α mRNA compared with the *Hexb* ^+/−^ *FcRγ^+/+^* mice (Fig. 5C).

### mRNA expression profile in the thymus of *Hexb* ^−/−^ *FcRγ* ^+/+^mouse

To further elucidate the molecular basis of thymic involution, we performed cDNA microarray analysis to identify the changes in gene expression that accompanied the involution of the thymus. Thymic gene expression of 15 week old *Hexb* ^+/−^ *FcRγ^+/+^* and *Hexb* ^−/−^ *FcRγ^+/+^* mice were examined with Affymetrix® Mouse Genome 430 2.0 Array containing 45101unique mouse gene sequences. The gene expression profile reported in this paper has been deposited in the Gene Expression Omnibus (GEO) database (http://www.ncbi.nlm.nih.gov/geo: accession no. GSE19641). 8018 probes were found to be relatively increased in the thymus of the *Hexb* ^−/−^ *FcRγ^+/+^* mice compared with that of the *Hexb* ^+/−^ *FcRγ^+/+^* mice. On the other hand, the expression of 7604 probes was relatively decreased. The cohort of up-regulated sequences was dominated by genes that play a role in the immune response. In addition, some of these genes are expressed in macrophage lineages such as macrophage expressed gene 1, and colony stimulating factor 2 receptor beta 1. Th2 cytokines were mostly upregulated in the *Hexb* ^−/−^ *FcRγ^+/+^* mice, although Th1 cytokines did not show this increase (Table 1). In addition, B cell-related genes such as CD19, CXCL13 were increased, whereas the T cell-related genes were mostly decreased, in *Hexb* ^−/−^ *FcRγ^+/+^* mice compared with *Hexb* ^+/−^ *FcRγ^+/+^* mice.

**Table 1:** mRNA expression profile in the thymus of the 15 week old *Hexb* ^−/−^ *FcRγ* ^+/+^ mouse.

Gene name (*Symbol*)	Base signal	Exp signal	Exp/Base signal Log ratio
***T lymphocyte***			
CD3 antigen, delta (*Cd3d*)	4510.3	1509.6	−1.6
CD4 antigen (*Cd4*)	1438.6	378.3	−1.8
CD8 antigen, alpha chain (Cd8a)	5002.2	235.3	−4.3
T-cell receptor alpha chain (*Tcra*)	55.1	15.1	−2.1
***Macrophage***			
Colony stimulating factor 2 receptor, beta 1 (*Csfr2b1*)	143	549.9	2.1
Macrophage ecpressed gene 1 (*Mpeg1*)	836.4	1905.2	1.3
Macrophage migration inhibitory factor (*Mif*)	2844.4	1810	−0.6
Monocyte to macrophage differentiation-asociated (*Mmd*)	328.9	592.2	0.8
***B lymphocyte***			
Early B-cell factor 1 (*Ebf1*)	17.9	144.5	3.1
CD79A antigen (*Cd79a*)	65.7	227.2	1.6
CD79B antigen (*Cd79b*)	155.7	407.5	1.4
CD19 antigen (*Cd19*)	6.7	125.3	3.8
CD38 antigen (*Cd38*)	167.5	497.9	1.9
Burkitt lymphoma receptor 1 (*Cxcr5*)	15.6	80.3	2.6
***Chemokine***			
Chemokine (C-X-C motif) ligand 13 (*Cxcl13*)	29	677.1	4.6
***Th2 cytokine***			
Interleukin 10 (*Il10*)	3.6	24.3	2.2
Interleukin 10 receptor, alpha (*Il10ra*)	49.2	118.4	1.2
Interleukin 10 receptor, beta (*Il10rb*)	330.8	742.2	1.3
Interleukin 13 receptor, alpha 1 (*Il13ra1*)	58.3	128.9	1.1
***Th1 cytokine***			
Tumor necrosis factor (*Tnf*)	14.1	50.4	1.9
Transforming growth factor, beta 1 (*Tgfb1*)	455	213	−0.9

The Affymetrix® Mouse Genome 430 2.0 Array was employed to identify changes in the gene expression profile in the thymus of a 15 week old of *Hexb* ^−/−^ *FcRγ* ^+/+^ mouse. ‘Signal’ represents a measure of the relative abundance of a particular transcript. ‘Exp/Base signal Log ratio’ denotes the change in the expression level for a transcript between Base group (15 week old *Hexb* ^+/−^ *FcRγ* ^+/+^ thymus) and the Experimental group (15 week old *Hexb* ^−/−^ *FcRγ* ^+/+^ thymus). This change is expressed as the log2 ratio. A log2 ratio of 1 is equivalent to a fold Change of 2. Representative genes that function in the immune system are listed.

### Marked increase in CXCL13 expression in the thymus of the *Hexb* ^−/−^ *FcRγ* ^+/+^ mouse

**Figure 6:**
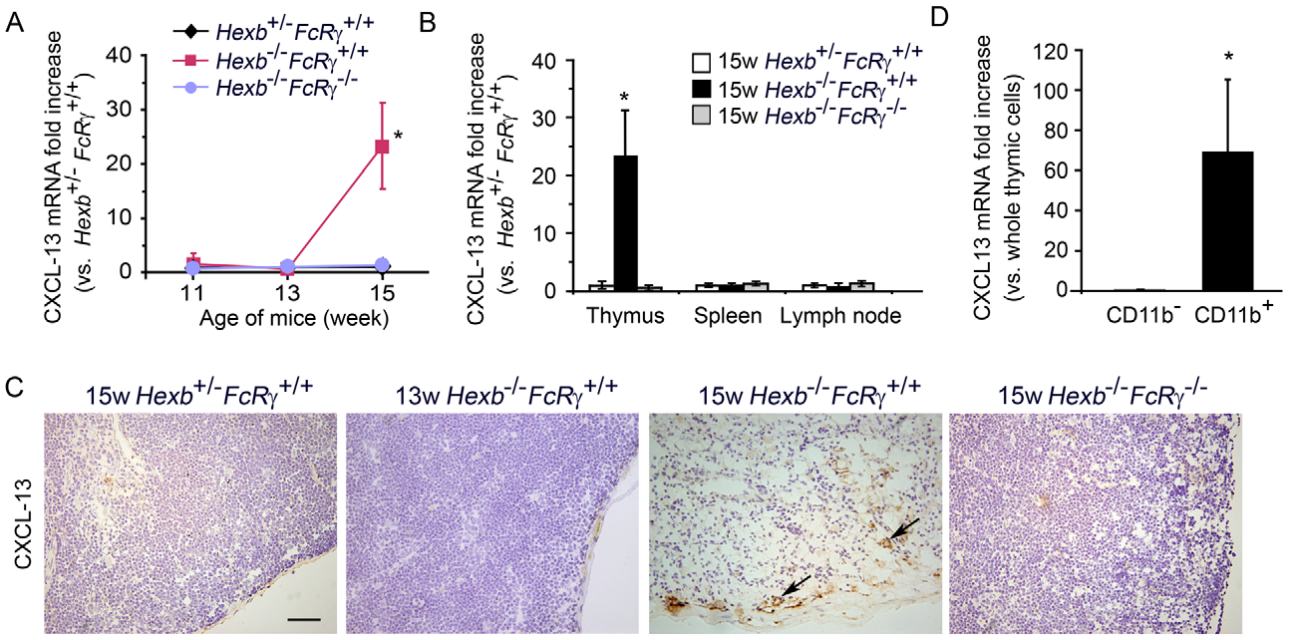
CXCL13 expression is markedly increased in the thymus of the 15 week old *Hexb* ^−/−^ *FcRγ* ^+/+^ mouse. (A) The CXCL13 mRNA levels in the thymus from each of the *Hexb* ^+/−^ *FcRγ^+/+^*, *Hexb* ^−/−^ *FcRγ^+/+^* and *Hexb* ^−/−^ *FcRγ* ^−/−^ mice at 11, 13 and 15 weeks of age were determined by real-time RT-PCR and compared with the levels in the *Hexb* ^+/−^ *FcRγ^+/+^* mouse (mean ± S.D.;*n* = 3–8). *P<0.05. (B) The CXCL13 mRNA levels in the thymus, spleen and lymph nodes from 15 weeks old *Hexb* ^+/−^ *FcRγ^+/+^*, *Hexb* ^−/−^ *FcRγ^+/+^* and *Hexb* ^−/−^ *FcRγ* ^−/−^ mice was also measured using real-time RT-PCR and compared with the levels in *Hexb* ^+/−^ *FcRγ^+/+^* mice (mean ± S.D.; *n* = 4–8). *P<0.05. (C) Thymus cryosections from 13 week old *Hexb* ^+/−^ *FcRγ^+/+^*, 15 week old *Hexb* ^+/−^ *FcRγ^+/+^*, *Hexb* ^−/−^ *FcRγ^+/+^* and *Hexb* ^−/−^ *FcRγ* ^−/−^ mice were stained with CXCL13 antibodies and detected using DAB. Arrows indicate CXCL13-positive cells. Scale bar, 50 µm. (D) Thymic cells from 15 week old *Hexb* ^−/−^ *FcRγ^+/+^* mice were cell sorted using MACS. The CXCL13 mRNA levels in the CD11b^+^ and CD11b^−^ cell populations are expressed relative to the normalized CXCL13 levels in whole thymic cells (mean ± S.D.; *n* = 4). *P<0.05.

Quantitative differences in CXCL13 expression in the thymus between *Hexb* ^+/−^ *FcRγ^+/+^*, *Hexb* ^−/−^ *FcRγ^+/+^* and *Hexb^−/−^FcRγ* ^−/−^ mice were assayed by real-time RT-PCR (Fig. 6A). There were no significant differences found in 11 or 13 week old *Hexb* ^+/−^ *FcRγ^+/+^*, *Hexb* ^−/−^ *FcRγ^+/+^* and *Hexb^−/−^FcRγ* ^−/−^ mice. However, an extremely high level of CXCL13 expression was observed in the thymus of 15 week old *Hexb* ^−/−^ *FcRγ^+/+^* mice compared with the normalized CXCL13 levels in *Hexb* ^+/−^ *FcRγ^+/+^* (23.3-fold) and *Hexb^−/−^FcRγ* ^−/−^ mice. The expression of several other cytokines/chemokines such as SDF-1 and TNF-α was not increased in *Hexb* ^−/−^ *FcRγ^+/+^* mice compared with *Hexb* ^+/−^ *FcRγ^+/+^* mice (data not shown). To assess whether the abnormal CXCL13 mRNA expression is thymus-specific, we quantified the CXCL13 mRNA levels in other lymphoid organs. The upregulation of CXCL13 was not found in the lymph nodes or spleen (Fig. 6B). In the thymus of 15week old *Hexb* ^−/−^ *FcRγ^+/+^* mice, the normal architecture was lost and CXCL13 was diffusely distributed throughout the entire region (Fig. 6C). Real-time RT-PCR analysis of magnetic cell sorted leukocyte subpopulations demonstrated that CXCL13 is highly expressed in thymic CD11b-positive cells, which are putatively regarded as macrophages, and also in dendritic cells obtained from 15 week old *Hexb* ^−/−^ *FcRγ^+/+^* mice, but not in CD11b-negative cells from these animals (Fig. 6D).

### The B1 and B2 cell populations are markedly increased in the thymus of *Hexb* ^−/−^ *FcRγ* ^+/+^ mouse

**Figure 7:**
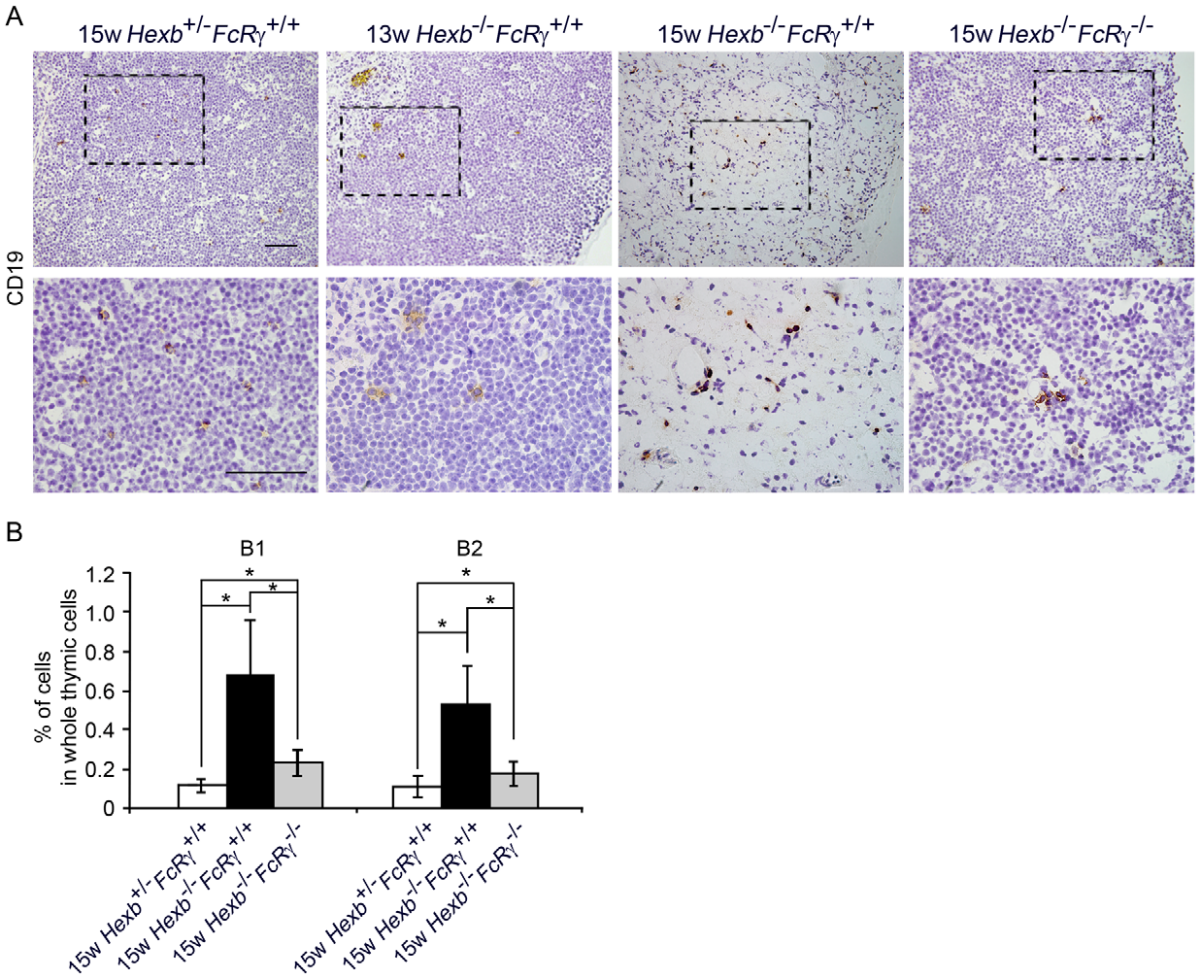
Significant increase in the B1 and B2 cell number in the thymus of 15 week old *Hexb* ^−/−^ *FcRγ* ^+/+^ mouse. (A) Thymus cryosections from 13 week old *Hexb* ^+/−^ *FcRγ^+/+^*, 15 week old *Hexb* ^+/−^ *FcRγ^+/+^*, *Hexb* ^−/−^ *FcRγ^+/+^* and *Hexb* ^−/−^ *FcRγ* ^−/−^ mice were stained with CD19 monoclonal antibodies and detected using the ABC method. The bottom panels show higher magnification images of the framed areas in the top panels. Scale bars, 50 µm. (B) Thymic cells from 15 week old *Hexb* ^+/−^ *FcRγ^+/+^*, *Hexb* ^−/−^ *FcRγ^+/+^* and *Hexb* ^−/−^ *FcRγ* ^−/−^ mice were stained with FITC-CD5, PE-B220 and PE-Cy5-CD19 antibodies and the percentages of the B cell subsets were determined by flow cytometry (mean ± S.D.; *n* = 6–9). *P<0.01.

The aberrantly high expression of CXCL13 in the thymus of mice with murine lupus plays a pivotal role in recruiting autoantibody-producing B cells. We evaluated whether B cells are increased also in the thymus of 15 week old *Hexb* ^−/−^ *FcRγ^+/+^* mice. By histological examination, we detected massive CD19-positive B cell infiltration and/or expansion in the thymus of the 15 week old *Hexb* ^−/−^ *FcRγ^+/+^* mice (Fig. 7A). In 13 week old *Hexb* ^−/−^ *FcRγ* ^+/+^ and 15 week old *Hexb* ^−/−^ *FcRγ* ^−/−^ mice, we did not see this B cell expansion. The percentages of CD19^+^/CD5^+^/B220^lo^ B1 cells were 0.12±0.04, 0.68±0.28 and 0.23±0.07% in the thymus of 15 week old *Hexb* ^+/−^ *FcRγ^+/+^*, *Hexb* ^−/−^ *FcRγ^+/+^* and *Hexb* ^−/−^ *FcRγ* ^−/−^ mice, respectively, and those of CD19^+^/CD5^−^/B220^hi^ B2 cells were 0.11±0.05, 0.53±0.19 and 0.17±0.06% respectively (Fig. 7B).

## Discussion

Sandhoff disease is a lysosomal storage disorder characterized by the absence of β-hexosaminidase and storage of GM2 ganglioside and related glycolipids. It is widely postulated that the CNS is the main pathological target of GM2 gangliosidosis. However, we have previously found that the production of autoantibodies plays an important role in the pathogenesis of neuropathy in Sandhoff disease [10]. In our present study, we observed a dramatic involution of the thymus in 15 week old *Hexb* ^−/−^ *FcRγ^+/+^* mouse. The thymus plays a crucial role in immune system homeostasis, and thymic abnormalities have been previously reported in many autoimmune diseases, such as myasthenia gravis [31], systemic sclerosis [32], in a mouse model of systemic lupus erythematosus [24], and multiple sclerosis [33]. Since autoimmunity is found also in SD, the contribution of thymus abnormalities to the pathogenesis of this disease seems plausible. We therefore tested for immunological alterations in the thymus of *Hexb* ^−/−^ *FcR^+/+^* mice during the development of mild to severe progressive neurologic disease and explored the relationship between thymic abnormalities and autoimmunity.

The thymus of the *Hexb* ^−/−^ *FcR^+/+^* mouse was found to be drastically involuted at the late stages, whereas this degree of involution was reduced in the *Hexb* ^−/−^ *FcRγ* ^−/−^ mouse (Fig. 1A, B), suggesting that this process is dependent on the FcR(chain. The involution itself was found to contain a markedly decreased percentage of immature CD4+/CD8+ T cells and significantly increased populations of CD4+/CD8- cells, CD4-/CD8+ T cells and CD4-/CD8- cells (Fig. 1C, top panel). The absolute number of each subpopulation was decreased, suggesting that the changes in cell number are strongly dependent on the decreased number of CD4+/CD8+ T cells, rather than the increase in CD4+/CD8- cells, CD4-/CD8+ T cells and CD4-/CD8- cells. In contrast, no differences could be clearly observed in the younger *Hexb^−/−^FcR^+/+^*mice. Zhou and co-workers have also shown in their previous study that the development of classical, naive, and memory CD4 and CD8 T cells is unaffected by a *Hexb* deficiency [34]. The authors did not however discuss the state of the thymus in later stages. In other LSDs, alterations in the last stage thymus have been previously reported in feline GM1 gangliosidosis [19], [20]. Flow cytometric analyses showed a marked decrease in the percentage of immature CD4^+^/CD8^+^ T cells and a significant increase in CD4^−^/CD8^+^ cells in GM1 mutant cats of greater than 210 days of age when compared with normal age matched cats [19]. Thymic involution has also been described in twitcher mice [21] and may therefore be a common pathological state among the LSDs.

We next examined for histological abnormalities in the thymus of *Hexb* ^−/−^ *FcRγ^+/+^* mice. In the 15 week old animals, the cellular density of the thymic cortices was found to be severely reduced as a result of a decreased number of CD4^+^/CD8^+^ cells (Fig. 2). However, numerous macrophages with a “foamy” appearance were found instead in this region. Some of these macrophages contained hematoxylin-positive particles in their cytoplasm, suggesting that the population of apoptotic thymic cells was rapidly increased and/or that the macrophages had a diminished phagocytic capacity. Importantly, a pathology similar to that evident in mouse models appears to exist also in human SD (Fig. 3). Moreover, atrophy of the thymus has previously been described in an autopsy case report by Takahashi *et al*. [35], and in a personal communication from Tatematsu *et al.* [36]. These observations suggest that thymic alterations occur in SD in both mouse and human via a common mechanism.

We next found in our mouse model that during thymic involution, nuclear particles in the cytoplasm of the macrophages, apoptotic thymic cells, and IgG deposition to the thymic cells were all increased, particularly in the cortex (Fig. 4). Most thymic lymphocytes are cleared by normal apoptotic processes but increased apoptosis can be observed in some animal models of LSD such as twitcher mice, and feline GM1 gangliosidosis. It is thought that the accumulation of GM2 induces neuronal cell death in GM2 gangliosidosis. Various extra-neural organs also contain many minor ganglioside components. In particular, the thymus has been found to have a very complex and characteristic spectrum of ganglioside species [37], [38]. Gadola et al have previously observed that glycolipids are stored in the thymus [22]. In addition, Zhou *et al.* have reported that GM1, GM2, GM3, and GT1b can induce apoptosis in thymocytes in vitro [18]. We have also confirmed in our experiments that GM2 enhances thymic cell death, resulting in the formation of *in vivo*-like T cell subpopulations *in vitro* (data not shown). However, we also found that the apoptosis and necrosis of thymic cells is decreased in 15 week old *Hexb* ^−/−^ *FcRγ* ^−/−^ mice, although the accumulation levels did not obviously differ from *Hexb* ^−/−^ *FcRγ^+/+^* mice (data not shown). This result suggests that the Fc receptor plays a role in thymic cell death in *Hexb* ^−/−^ *FcRγ^+/+^* mice. We have previously detected the presence of anti-GM2 and anti-GA2 autoantibodies in end stage *Hexb* ^−/−^ *FcRγ^+/+^* mice [10] and also other autoantibodies such as anti-ssDNA (unpublished data), which suggests the presence of polyclonal antibodies in *Hexb* ^−/−^ *FcRγ^+/+^* mice. In our present study, we observed the deposition of IgG on T cell membranes (Fig. 4E). These findings suggest that an anti-T cell autoantibody is produced during the last stages in *Hexb* ^−/−^ *FcRγ^+/+^* mice and that an IgG-FcR mediated pathway plays a role in the thymic involution seen in *Hexb* ^−/−^ *FcRγ^+/+^* mice.The thymus from *Hexb* ^−/−^ *FcRγ^+/+^* mice older than 15 weeks showed a marked increase in the percentage of macrophages, particularly in the cortex (Fig. 5A, B). Additionally, these macrophages were found to contain many nuclear particles in the cytoplasm. These results are consistent with the findings of previous histological experiments using H&E, TEM and TUNEL analyses. Mip-1α which is an important molecule in the pathogenesis of *Hexb* ^−/−^ mice [39], is upregulated in the thymus of 15 week old *Hexb* ^−/−^ *FcRγ^+/+^* mice. The evidence suggests therefore that the increase in the number of macrophages is partly due events that occur peripherally to the thymus.

To better understand the molecular basis of thymic involution, we performed cDNA microarray analysis to identify the changes in gene expression that accompanied involution in this gland. The results revealed an upregulation of immune response associated genes dominated by macrophages. A question that then emerged was how the macrophages were activated at this stage. It is very possible that enhanced cell death and/or the FcRγ dependent signaling pathway is involved in the mechanism. Further analysis is needed to determine whether the FcRγ pathway does contribute to gene expression changes in the thymus. On the other hand, prior to autoantibody production, FcRγ independent pathways may induce thymic alterations. In this regard, previous reports have indicated that in the CNS of *Hexb* ^−/−^ mice, the accumulation of undegraded glycolipids in glial cells might be a trigger of pro-inflammatory cytokine/chemokine production [8], [40]. It has also been reported that the induction of Mip-1α might coincide with the accumulation of *N*-acetylhexosaminyl glycoconjugates due to a *Hexb* deficiency in glial cells [31]. It is also known that macrophages accumulate considerable levels of glycolipids within late endosomes/lysosomes during the onset of Sandhoff disease [22].

We detected many vacuoles in the cytoplasm of thymic macrophages, which we speculated to be lysosomal storage (Fig. 4A). Moreover, Periodic acid-Schiff (PAS) positive glycoconjugates were found to accumulate in the cytoplasm of thymic macrophages (data not shown). Previously, Kawane *et al.* found that DNaseII-deficient mice developed a rheumatoid arthritis-like disease [41] and concluded that, if macrophages cannot degrade mammalian DNA from erythroid precursors and apoptotic cells, they produce TNF-α, which activates synovial cells to produce various cytokines, leading to the development of chronic polyarthritis. DNaseII-deficient mice and *Hexb* ^−/−^ *FcRγ^+/+^* mice have a common phenotype in which undegraded substances accumulate in the macrophages. It has been postulated that a Toll-like receptor independent pathway underlies the molecular mechanism by which cells sense the DNA that has escaped from degradation and initiate an autoimmune response. Other recent reports suggest also that the NALP3 inflammasome senses lysosomal damage as an endogenous ‘danger’ signal and thus induces inflammation in many diseases [42]–[46]. The accumulation of glycolipids within macrophages, and also following the activation of the inflammasome, may therefore be related to the gene expression changes we observe in the thymus of *Hexb* ^−/−^ *FcRγ^+/+^* mice.

CXCL13, an upregulated gene in end stage *Hexb* ^−/−^ *FcRγ^+/+^* mice, was found to be specifically expressed in the thymus, and B1 cells were also detected at increased levels in the thymus (Fig. 6A, B, 7). High expression of CXCL13 has been found previously in many autoimmune diseases, such as myasthenia gravis [31], multiple sclerosis [33], and in a model mouse of SLE [26], and is considered to be associated with autoantibody production. We have shown also in our previous study that a significant elevation of serum antibody levels occurs in the terminal stages of *Hexb* ^−/−^ *FcRγ^+/+^* mice of more than 14 weeks of age [10]. Thus, a high expression of CXCL13 and infiltration of B1 cells in *Hexb* ^−/−^ *FcRγ^+/+^* mice may be a reflection of autoantibody production.

Based on our current results, we propose a mechanism by which thymic alterations occur in *Hexb* deficient mice as follows. The apoptosis of immature T cells occurs normally in *Hexb* ^−/−^ *FcRγ^+/+^* mice and these apoptotic T cells are engulfed by macrophages. However, macrophages cannot degrade the glycolipids from apoptotic T cells in these animals because of their *Hexb* deficiency, and thus accumulate these molecules in lysosomes. The macrophages are then activated and produce CXCL13, which promotes chemotaxis toward B1 cells and thus leads to the development of autoimmunity. Once autoantibodies against T cells are produced, the storage of T cell-derived glycolipids by macrophages is enhanced via the autoantibody-dependent phagocytosis of T cells. This undesired loop results in a disrupted immune system and in the production of autoantibodies against neuronal cell antigens or neuronal cell - T cell common antigens such as GA2. Deletion of *FcRγ* may thus prevent thymic involution by blocking the autoantibody dependent phagocytosis of T cells and suppressing specific gene expression. A reduction of the serum titer against GM2 and GA2 in *Hexb* ^−/−^ *FcRγ* ^−/−^ mice [10] might be related to thymic integrity. Further studies involving other factors, such as functional testing of single lymphocytes and innate immunity, will be needed to further our understanding of autoimmunity in GM2 gangliosidoses.

## Materials and Methods

### Animals

All mice used in this study were bred and housed under specific pathogen-free (SPF) conditions. All mouse experiments were approved by the Animal Committee at Yokohama City University. SD model mice (*Hexb^−/−^* mice; C57BL/6×129/Sv background) were kindly provided by R. L. Proia (NIH, Bethesda, MD) and were bred in a closed colony over 30 generations to inbreed for C57BL/6- and 129/Sv-derived genes [4]. *FcRγ* gene deficient *Hexb* ^−/−^ mice (*Hexb* ^−/−^ *FcRγ* ^−/−^) were generated by described as previously [10]. Each experiment utilized tissue from *Hexb* ^+/−^ *FcRγ^+/+^*, *Hexb* ^−/−^ *FcRγ^+/+^* and *Hexb* ^−/−^ *FcRγ* ^−/−^ mice. All mice were euthanized by inhalation of sevoflurane and the thymus in each case was collected aseptically for further analysis.

### Human thymus

A hematoxylin and eosin (H&E) stained slide of a human thymus, dissected from an 11-month-old male with SD who was autopsied at Kobe Children's Hospital [47], was kindly loaned by Dr. H. Itoh.

### Histopathology

The mice were perfused with 10% buffered formalin (Sigma-Aldrich Corp. St. Louis, MO) and processed for paraffin embedding. Deparaffinized and rehydrated sections (4 µm) were stained with H&E or used for immunostaining. Apoptosis was detected via the *in situ* terminal deoxy nucleotidyl transferase-mediated dUTP nick end labeling (TUNEL) method using an ApopTag® kit (Millipore, Billerica, MA). For staining ionized calcium binding adaptor molecule 1 (Iba-1), sections were treated with antigen retrieval reagent and then incubated with rabbit anti-Iba-1 antibody (Wako Pure Chemical Industries, Ltd., Osaka, Japan) overnight at 4°C. The washed sections were incubated with biotinylated anti-rabbit IgG antibody (Nichirei, Tokyo, Japan) and subsequently incubated with horseradish peroxidase (HRP) labeled streptavidin (Nichirei). The peroxidase reaction was visualized by diaminobenzidine (DAB) and hydrogen peroxide. For frozen sections, the thymus of each mouse were embedded in OCT compound (Sakura Finetek, Torrance, CA) and quickly frozen in dry ice. For the staining of CXCL13, frozen sections (7 µm) were stained with goat anti-CXCL13 pAb (R&D Systems, Inc., Minneapolis, MN), followed by HRP labeled goat anti-rat IgG (Nichirei). For the staining of CD19, frozen sections (7 µm) were stained with Rat anti-CD19 mAb (6D5; Chemicon International, inc., Temecula, CA), followed by HRP labeled goat anti-rat IgG (Nichirei). For Transmission electron microscopy (TEM), thymic tissues was prefixed with 2.5% glutaraldehyde (Merck & Co., Inc., Whitehouse Station, NJ) and postfixed with 1% osmium tetroxide (Wako Pure Chemical). The samples were embedded in Epon 812 resin (TAAB Laboratories, Aldermaston, Berkshire, UK). Ultrathin sections were stained with uranyl acetate and lead citrate (Wako Pure Chemical). Samples were observed with an H-7500 electron microscope (Hitachi, Tokyo, Japan).

### Flow cytometry

The remaining thymus samples were gently mechanically dispersed into PBS containing 3% fetal bovine serum (staining buffer) and filtered through a BD Falcon™ Cell Strainer (70 µm pore size; BD Biosciences, Franklin, NJ) to produce single cell suspensions and washed twice with staining buffer. Aliquots of 1×10^6^ thymic cells were incubated with PE-labeled anti-CD4 (GK1.5; eBioscience, Inc., San Diego, CA) and FITC-conjugated anti-CD8 (53-7.6; eBioscience) mouse monoclonal antibodies (mAbs). For three color staining, thymic cells were stained with PE-labeled anti-B220/CD45R (RA3-6B2; BD Biosciences) and FITC-conjugated anti-CD5 (53-7.3RRH; eBioscience) and PE-Cy5 conjugated CD19 (MB19-1; eBioscience) mAbs. For the staining of thymic macrophages, the thymic cells were stained with anti-Iba1 (Wako Pure Chemical), followed by Alexa Fluor ®-488 labeled anti-rabbit IgG (H+L) (Invitrogen, Carlsbad, CA). For the detection of autoantibody deposition on T cells, thymic cells were stained with Goat anti-mouse IgG (Southern Biotech, Birmingham, AL) followed by Alexa Fluor®-488 labeled Donkey anti-Goat IgG (H+L) (Invitrogen) and PE labeled anti-TCRβ (H57-597; eBioscience). Flow cytometric analysis was carried out using FACScan™ with CELLQuest™ software (BD Biosciences) or with FACS Canto II™ with FACSDiva™ software (BD Biosciences).

### cDNA microarray

Pooled thymic RNA samples were obtained from two 15 weeks old Hexb^−/−^ mice and two strain-matched, 15 weeks old Hexb^+/−^mice. cDNA were synthesized by GeneChip T7-Oligo(dT) Promoter Primer Kit (Affymetrix, Inc, Santa Clara, CA) and TaKaRa cDNA Synthesis Kit (TaKaRa Bio Inc, Shiga, Japan) from 10 µg total RNA. Biotinylated cRNA were synthesized by IVT Labeling Kit (Affymetrix). Following fragmentation, 10 µg of cRNA were hybridized for 16 hr at 45°C on GeneChip Mouse Genome 430 2.0 Array (Affymetrix) containing 45101 unique mouse genes. GeneChips were washed and stained in the Affymetrix Fluidics Station 450. GeneChips were scanned using GeneChip Scanner 3000 7G. Single Array Analysis were calculated by Microarray Suite version 5.0 (MAS5.0) with Affymetrix default setting and global scaling as normalization method. The trimmed mean target intensity of each array was arbitrarily set to 500. Signals were calculated using the One-Step Tukey's Biweight Estimate which yields a robust weighted mean. The signal log ratio was computed using a one-step Tukey's Biweight method by taking a mean of the log ratios of the probe pair intensities across the two arrays. All data is MIAME compliant and that the raw data has been deposited in GEO.

### Real-time RT-PCR

Total RNA was purified with TRIzol Reagent (Invitrogen). To determine the high CXCL13-expressing cells, a magnetic cell sorting system was used for the RNA preparation. Briefly, a 1×10^6^ thymic cell suspension from 15 week old *Hexb* ^−/−^ *FcRγ^+/+^* mice was stained with CD11b MicroBeads (Miltenyi Biotec, Bergisch Gladbach, Germany) and separated using an MS Column (Miltenyi Biotec), and a MiniMACS® Separator (Miltenyi Biotec). cDNA was synthesized from total RNA by reverse transcription using a commercial cDNA synthesis kit (TaKaRa Bio Inc). cDNA synthesized from 500 ng of total RNA was used as the template in each reaction. The relative gene expression levels were determined using the SYBR® *Premix Ex Taq*™ II (TaKaRa Bio Inc). The primer sets for ribosomal protein s18 (Rps18) and tumor necrosis factor-alpha (TNF-α) were obtained from TaKaRa Bio. The primer set for macrophage inflammatory protein-1 alpha (Mip-1α) has been described previously [31]. The primers used to amplify Chemokine (C-X-C motif) ligand 13 (CXCL13) are 5′-TCTCTCCAGGCCACGGTATTCT-3′ (forward, F) and, 5′-ACCATTTGGCACGAGGATTCAC (reverse, R) and for stromal cell-derived factor 1 (SDF-1) are 5′-GAGCCAACGTCAAGCATCTG-3′ (F) and 5′-CGGGTCAATGCACACTTGTC-3′ (R). Rps18 was amplified in each reaction simultaneously as a standard control. The fluorescence changes from each well were monitored using an ABI PRISM® 7500 Sequence Detection System (Perkin Elmer, Inc, Waltham, MA).

### Statistical analysis

Statistical analysis was performed using the Student's *t* test. A 95% confidence limit was taken as significant.

## Supporting Information

Figure S1 Immunofluorescent analysis of GA2 and thymocyte, for each thymus derived from *Hexb* ^+/−^ *FcRγ* ^+/+^, *Hexb* ^−/−^ *FcRγ* ^+/+^ and *Hexb* ^−/−^ *FcRγ* ^−/−^ mice. Frozen section of the thymus from 15 week old *Hexb* ^+/−^ *FcRγ* ^+/+^, *Hexb* ^−/−^ *FcRγ* ^+/+^ and *Hexb* ^−/−^ *FcRγ* ^−/−^ and 13 week old *Hexb* ^−/−^ *FcRγ* ^+/+^ mice were labeled GA2 with Alexa Fluor®-488, CD4 and CD8 with Alexa Fluor®-594, and nuclear with Hoechst 33258, respectively. Arrows indicate the deposition of GA2 in the cytosol of the CD4/8 negative cells. Scale bar, 20 μm.
(0.43 MB PDF)
