# Supplementary figures and images for: Thymic Alterations in GM2 Gangliosidoses Model Mice
Source: PLoS One. 2010 Aug 10;5(8):e12105. doi: 10.1371/journal.pone.0012105 (PMC2938369; doi:10.1371/journal.pone.0012105)

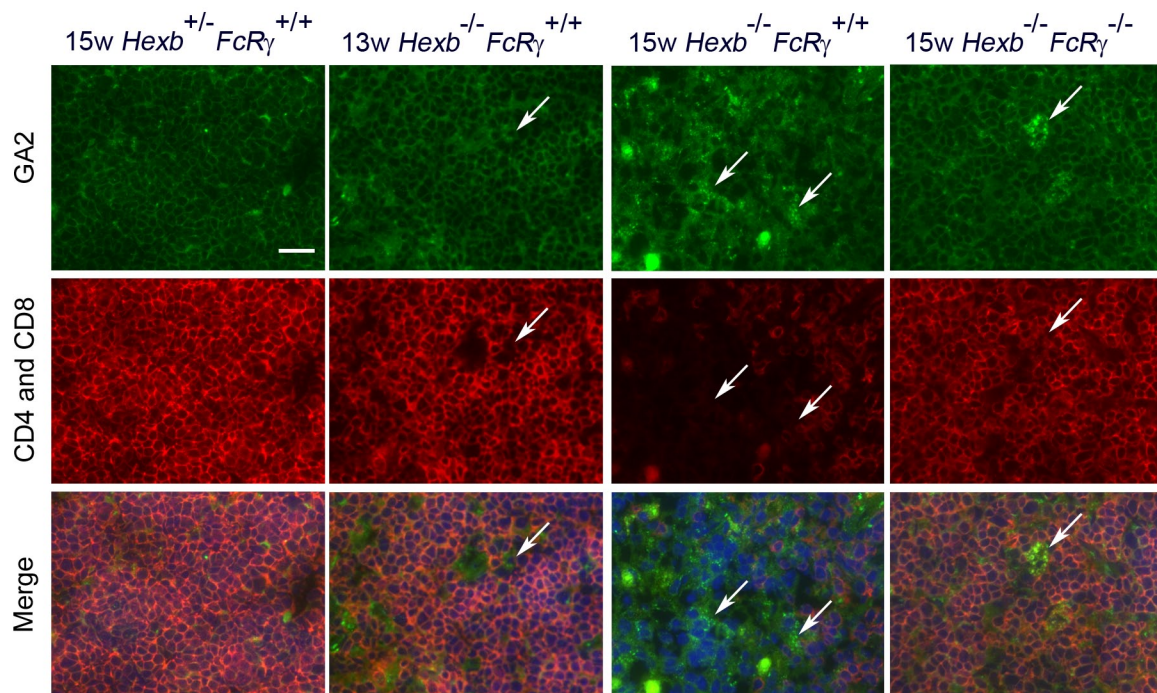

Supplement: Figure S1 — Immunofluorescent analysis of GA2 and thymocyte, for each thymus derived from Hexb +/− FcRγ +/+, Hexb −/− FcRγ +/+ and Hexb −/− FcRγ −/− mice. Frozen section of the thymus from 15 week old Hexb +/− FcRγ +/+, Hexb −/− FcRγ +/+ and Hexb −/− FcRγ −/− and 13 week old Hexb −/− FcRγ +/+ mice were labeled GA2 with Alexa Fluor®-488, CD4 and CD8 with Alexa Fluor®-594, and nuclear with Hoechst 33258, respectively. Arrows indicate the deposition of GA2 in the cytosol of the CD4/8 negative cells. Scale bar, 20 μm. (0.43 MB PDF) [file pone.0012105.s001.pdf]
